# Supplementary material for: Overexpression of Pear (Pyrus pyrifolia) CAD2 in Tomato Affects Lignin Content
Source: Molecules. 2019 Jul 17;24(14):2595. doi: 10.3390/molecules24142595 (PMC6680972; doi:10.3390/molecules24142595)
Supplement: Supplementary file 1 [file molecules-24-02595-s001.pdf]

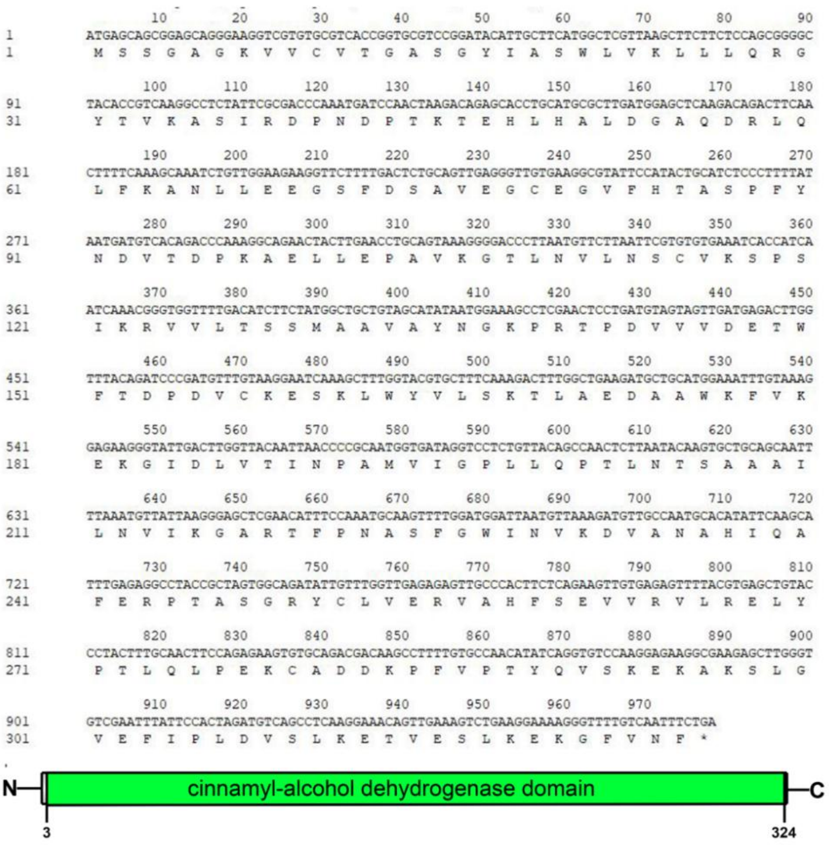

Figure S1. PpCAD2 sequence analysis. Upper row: Nucleic acid and amino acid sequences of PpCAD2. Bottom row: schematic representation of PpCAD2 proteins with its respective conserved domains.

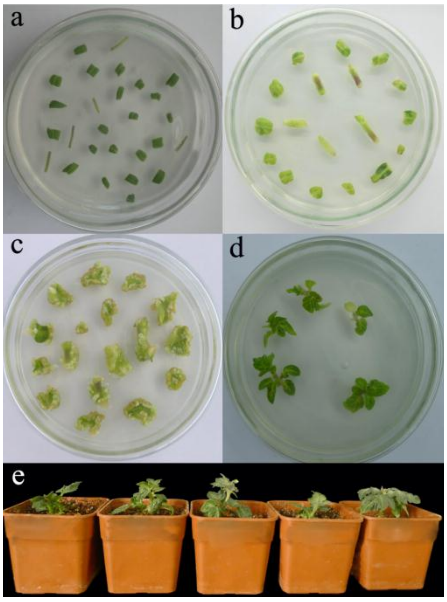

Figure S2. Generation of transgenic tomato 'Micro-Tom'. a: Leaf (cotyledon) discs before Agrobacterial inoculation; b: Co-culture; c: Callus formation; d: germinated plantlets with shoots; e:

transgenic plants after transfer to soil.

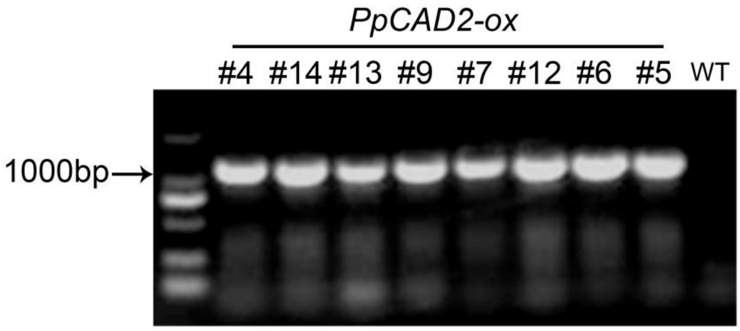

Figure S3. PCR identification of transgenic ‘Micro-Tom’ plants with the PpCAD2 constructs.
